# Comparing and Contrasting Professional Identity Formation Among Health Professional Students

**DOI:** 10.7759/cureus.65577

**Published:** 2024-07-28

**Authors:** Varun S Rachakonda, Sunaina Addanki, Hazem Nasef, Vijay Rajput

**Affiliations:** 1 Medical Education, Nova Southeastern University Dr. Kiran C. Patel College of Allopathic Medicine, Fort Lauderdale, USA

**Keywords:** healthcare professional, teaching competencies, health professional's education, interprofessional education, professional identity formation

## Abstract

This project aims to review and compare the professional identity formation (PIF) of medical, dental, nursing, and pharmacy students while analyzing the role of interprofessional education (IPE) in this journey. Our medical research librarian conducted a literature review. Papers were selected based on the inclusion criteria developed by authors for PIF and IPE topics, which were then stratified for each health program of interest: medicine, dentistry, nursing, and pharmacy. The IPE core competencies were analyzed to understand the effect of IPE on each respective group of health professional students. Among all four major health professions, trust, collaboration, responsibility, accountability, communication, and empathy are key values within PIF. Trust, collaboration, and empathy were also regarded as core values in developing professionalism. Medical and dental students placed greater emphasis on responsibility and accountability regarding PIF. IPE played a crucial role in PIF for all students as values, teamwork, roles, and responsibilities were emphasized among each healthcare discipline of interest. This review provides significant information regarding which characteristics are emphasized for professional development across healthcare training programs. Future research to explore how certain characteristics and values influence healthcare as a whole is crucial. Investigating various influences on PIF outcomes is warranted for enhancing professional training programs and promoting interprofessional collaboration for better healthcare delivery.

## Introduction and background

Healthcare professional students embark on the journey to embed core values and virtues throughout their classroom and experiential learning. Each professional should display and carry those virtues and values throughout the career of their respective profession. This is known as professionalism [1]. Professionalism is an outward expression of a community's norms. On the other hand, professional identity formation (PIF) is an internalization of those norms such that one thinks, acts, and feels like a member of that community. The process of changing one's values in hopes of identifying better with a specific health profession is known as the PIF [2]. PIF is a complex, continuous, and multifaceted process that healthcare professional students undergo throughout their training [3]. Literature has shown that students' immersive experiences with patients, peers, healthcare professionals, and society during their years of training play a critical role in a student’s PIF [4]. To achieve PIF, various values are cultivated during their training in health professional schools [4].

Interprofessional education (IPE) is a collaborative model where students from various health professions learn about each other's roles, values, and ethics, gaining insight into their own responsibilities and limitations [5]. It focuses on four core competencies: values and ethics, roles and responsibilities, communication, and teamwork [5]. Different health professional schools emphasize various competencies throughout their IPE efforts.

This paper attempts to compare and contrast the unique process of PIF among respective health professionals, ranging from medical, dental, nursing, and pharmacy students. It also aims to understand how IPE provides value in the journey of PIF and which core values play a key role.

## Review

Methods

Study Selection

A literature search was conducted by the medical research librarian utilizing the following keywords: “professionalism,” “professional identity formation,” “medical student,” “nursing,” “dental,” and “pharmacy.” The search for articles was conducted using the PubMed and Google Scholar databases. The search covers all relevant articles and studies from the past 20 years to the present, ensuring a comprehensive understanding of the topic's development and current state. The literature evaluated the development of professional identity among healthcare professional students. The selection of the aforementioned professional schools was based on published literature evaluating similar outcomes. This initial search yielded 3,070 articles, which underwent further screening by all authors based on the exclusion and inclusion criteria. Duplicate articles were removed. Articles were excluded if they were written in a language other than English, were not relevant to the subject matter, or were not accessible by text, resulting in 258 articles. After a full-text review, the inclusion criteria were applied, focusing on peer-reviewed articles in English that addressed PIF or IPE across health professional schools, yielding 15 articles. These articles were then categorized based on specific professional schooling groups.

Outcomes of Interest

The literature was analyzed to identify key insights and shared PIF competencies among all the groups. The specific values (trust, collaboration, responsibility, accountability, communication, and empathy) were selected based on the amount of substantial literature available across the health professional schooling groups. Importance ratings for each competency were completed by contextual and thematic analysis of the articles. Frequency counts of each competency within the articles were identified, along with a discussion of the competency within the discussion and a conclusion for each source. Frequency counts and mentioning topics in the discussion and conclusion were identified as objective measures of significance for each competency.

A similar approach was used to assess the significance of IPE on PIF. Articles were first grouped according to specific professional schooling groups. The analysis focused on the importance of IPE and its core competencies (values and ethics, roles and responsibilities, communication, and teamwork) based on the Interprofessional Education Collaborative (IPEC) core competencies [2]. To evaluate each competency's significance, we conducted the same contextual and thematic analysis, including frequency counts of IPE mentions and examining how IPE concepts were discussed in the articles' discussions and conclusions sections. This analysis aimed to clarify how IPE core competencies contribute to PIF within different professional schooling groups.

Results

Medical Students

Five studies evaluated the process of PIF among medical students. Blackall et al. discussed the American Board of Internal Medicine's definition of professionalism, which consists of a list of elements including altruism, accountability, excellence, duty, honor and integrity, and respect for others [6]. Al-Temimi et al. evaluated the core competencies of the Accreditation Council for Graduate Medical Education, with professionalism being emphasized as one of its six core competencies [7]. Findyartini et al. described that medical students conceptualize the development of a professional identity with internal and external factors [3]. Internal factors include intrinsic motivation, values, individual circumstances, ability, and traits such as coping with challenges, asking critical questions, and practicing work-life balance. On the other hand, extrinsic factors such as curriculum, education management system, learning environment, and expectations from faculty and community also strongly affect PIF. This study also described students’ beliefs regarding burdensome assignments and monotonous teaching hindering professional development, while explicit teaching and collaborative learning activities strengthen identity formation [3].

Furthermore, Findyartini et al. discussed the Kegan Model where PIF in medical education is classified into six different stages [3]. These stages include incorporation, impulsion, imperial, interpersonal, institutional, and inter-individual stages [3]. The learners undergo stages two to four, emphasizing impulsion in interpersonal stages throughout schooling [3]. The imperial stages occur when students identify professional rules without self-reflection. After students advance to the institutional stage, they understand how relationships differ in values and expectations. Finally, students begin to internalize values, including professionalism, in the institutional stage [3]. The assessment of professionalism among medical students occurs in a variety of ways. Benbassat described peer assessments, direct observations during patient encounters, assessments of patient care, and multiple-choice situational judgment tests as a form of assessment [8]. Bray et al. explained that within the clerkship phase of the medical curriculum, professionalism is ensured by using Entrustable Professional Activities (EPA) as a framework. These activities include applying knowledge, providing feedback, and collaborative learning and are evaluated by residents, fellows, and attendings [9].

Dental Students

Four studies evaluated the process of PIF among dental students. Zijlstra-Shaw et al. defined PIF among dental students as how one reflects responsibility and accountability [10]. This study described how accreditation bodies of dental programs divided professionalism into implicit and overt aspects. Implicit aspects included understanding one's ability, understanding others, trustworthiness, an ability to relate to context, and reflection. On the other hand, overt aspects of professionalism include commitment, accountability, and responsibility [10]. Kwon et al. analyzed five different components contributing to PIF among dental students. These include domain-specific self-efficacy, mentors, peer socialization, learning environment, and reflection [11]. Mei et al. described how dental students prioritized domain-specific self-efficacy in their development of PIF [12]. Zijlstra-Shaw et al. identified reflection and feedback as key components of the development of PIF [10]. In another study by Zijlstra-Shaw et al., the Assessment of Dental Student Professionalism System (ADSPS) was described through the use of student self-assessments, faculty assessments that combine the views of all supervisors, and an appraisal discussion [13]. The assessment rubric identifies commitment, consideration, responsibility, trustworthiness, ability to instill trust, respect for rules, reflection of one's actions, and understanding of one's abilities. This study mentions through engaging in discussions with faculty involving one's ADSPS, dental students better understand their expected professional identity, which ultimately affects their PIF [13].

Pharmacy Students

Two studies evaluated the process of PIF among pharmacy students. According to a study conducted by Quinn et al., the process of PIF in pharmacy students occurs in four stages: reflection, selection of attributes, professional socialization, and perception of role [14]. However, it is quite difficult to complete all four stages during their time in pharmacy school [14]. The authors identified attendance in classroom activities as the most crucial factor in PIF for pharmacy students. Active participation among students was more likely to have experiences and interactions with learners and faculty of different backgrounds, allowing them to complete these four stages of PIF development [14]. Pharmacy students attributed importance are confidentiality, competence, and communication for their PIF. They emphasized both individual thinking and collaboration with other health professionals when making clinical decisions [14]. A study conducted by Kennie-Kaulbach et al. found that patient experience played a crucial factor in PIF among first-year pharmacy students [15]. After the patient experience, the role modeling of their specific preceptor was instrumental in their PIF [15].

Nursing Students

Four studies were reviewed for the process of PIF among nursing students. A study conducted by Bijani et al. discussed how nursing students identified their nursing instructors as one of their first sources of professional values [16]. These instructors were deemed pivotal for disseminating nursing and ethical values to their students [16]. Poorchangizi et al. discussed the assessment of nursing students' perception of professional values being caring, activism, trust, professionalism, and justice [17]. In a study conducted by Wang et al., the professional identity of nurses depends on their work environment and associated factors, which either limit or expand the role and authority of nursing professionals [18]. This emphasized early exposure to a clinical setting for the first year of nursing school to spark the PIF and the cultivation of empathy [18]. Cao et al. found that nursing instructors who embody professional values within a clinical setting early in nursing education provide the most optimal environment for cultivating a professional identity [19]. These environments in conjunction with unique experiences challenging core principles of a nurse's identity, such as serving as a nurse in a prison setting, were noted to solidify the professional identity of a nurse within the challenging work environment [19].

Comparative Analysis of PIF Among Health Professionals

We found that trust, collaboration, responsibility, accountability, communication, and empathy were the core values that contribute to PIF among all the healthcare professions of interest. However, there was a different degree and level of emphasis placed on each of these values by the healthcare professionals during their training. All four professional schools put a high emphasis on collaboration. Dentists, physicians, and nursing schools tend to put a major emphasis on trust and empathy. Physicians, pharmacists, and nurses tend to emphasize communication more than dentists. Physicians and dentists placed a higher emphasis on accountability in comparison with pharmacists and nurses (Table 1).

**Table 1:** Shared values of professional identity formation among medical, dental, pharmacy, and nursing students. The emphasis placed on each of these qualities within each curriculum was estimated on a scale of one to four, with four indicating the greatest amount of importance and one indicating less importance.

Values	Medical	Dental	Pharmacy	Nursing
Trust	✓✓✓✓	✓✓✓✓	✓✓	✓✓✓✓
Collaboration	✓✓✓✓	✓✓✓✓	✓✓✓✓	✓✓✓
Responsibility	✓✓✓✓	✓✓✓✓	✓✓	✓✓
Accountability	✓✓✓✓	✓✓✓✓	✓	✓✓
Communication	✓✓✓✓	✓✓	✓✓✓✓	✓✓✓
Empathy	✓✓✓✓	✓✓✓✓	✓	✓✓✓✓

Comparative Analysis of IPE Core Values Among Health Professionals

Physicians, dentists, pharmacists, and nurses all gave high importance to the values of ethics and also teamwork. Dentists and pharmacists put a higher emphasis on roles and responsibilities in their specific occupation after working in IPE sessions. Physicians, dentists, and pharmacists placed an increased emphasis on communication, with physicians putting the highest emphasis, after working in IPE sessions (Table 2).

**Table 2:** Level of emphasis on each IPE core competency among medical, dental, pharmacy, and nursing students. The emphasis placed on each of these qualities within each curriculum was estimated on a scale of one to four, with four indicating the greatest amount of importance and one indicating less importance.

Core Competencies	Medical	Dental	Pharmacy	Nursing
Values and ethics	✓✓✓✓	✓✓✓	✓✓✓	✓✓✓
Roles and responsibilities	✓✓	✓✓✓✓	✓✓✓✓	✓✓✓
Communication	✓✓✓✓	✓✓✓	✓✓✓	✓
Teamwork	✓✓✓	✓✓✓✓	✓✓✓	✓✓✓

Discussion

Among these various health professions, there are common shared values regarding the development of PIF representing their developmental role within healthcare. Based on analysis, medical and dental schools have shown the most common shared values when considering aspects of PIF. Analysis regarding specific aspects of PIF among all four health professional schools yielded crucial results.

Trustworthiness was identified as a common thread of PIF that dentists, nurses, and physicians prioritized when defining, developing, and assessing professionalism during their training. This quality is a foundational element of all four healthcare professional and patient relationships as it may provide patients with more reassurance and confidence in their treatment. Trustworthiness also adds to maintaining high ethical standards and appropriate professional boundaries, which are essential to forming a professional identity and ensuring that there is reliable patient care [2]. In both dental education and medical training, the process of emphasizing trustworthiness was seen as crucial due to students seeing this core value being reinforced by their senior professionals [20]. This core value is strengthened as senior professionals consistently model ethical behavior, reliability, and integrity. Their actions help instill these traits in students and foster a trusting atmosphere in the healthcare setting.

Among physicians, pharmacists, and dentists, collaboration was a common value in PIF. When professionals from these diverse fields can share their expertise and work together to treat a patient, greater patient outcomes prevail [21]. Additionally, collaboration among various professions helps individuals grow their knowledge and skill set. For physicians, collaboration helps integrate various perspectives in patient care. Research has shown that physicians collaborating with pharmacists and other healthcare providers is linked to improved patient outcomes [22]. For dentists, collaboration is crucial for comprehensive patient care. These collaborations with other healthcare providers are mostly used in areas such as perioperative oral management and dysphagia rehabilitation [23]. Studies have shown that for pharmacists, collaboration with other healthcare providers helps pharmacists understand their roles more clearly and strengthens their sense of professional identity [22].

When comparing medical and dental schools, accountability seems to play a bigger role compared to nursing and pharmacy when defining professionalism. A reason why accountability plays an integral role in nursing is because it is intertwined with trust, professionalism, and ethical practice. Studies show that in nursing students and nurses, accountability is a core foundation because of their expectation to adhere to strict ethical guidelines, provide high-quality patient care, and decision making in clinical settings [24]. For pharmacists, accountability plays a big role in professionalism because when dispensing medications to patients, they need to take extra care when counseling and educating patients on how to or when to take medications. A study conducted by Kellar et al. found that the professional identity in pharmacy is significantly shaped by aspects of accountability, impacting how pharmacists view their roles and responsibilities in the field of healthcare [25].

Furthermore, nursing and medical schools prioritized the importance of empathy when assessing professionalism. As both professions require empathy to make patients feel heard and understood, this quality enhances patient satisfaction and overall health outcomes. As both professions require empathy to make patients feel heard and understood, this quality enhances patient satisfaction and overall health outcomes. However, on a deeper level, nurses and physicians tend to spend the most amount of time with patients in the hospital setting. For physicians, empathy is important for patient communication, trust, and satisfaction. Empathetic physicians are better able to understand patients' concerns and fears. This is crucial for making accurate diagnoses and effective treatment plans. Research also demonstrates that fostering empathy in medical students can positively correlate with their sense of PIF scores [26]. For nurses, empathy fosters a therapeutic relationship with the patient that goes beyond clinical treatment. This connection is essential for patient trust and compliance, which are vital for effective care and recovery [24].

Finally, medical students and pharmacy students shared the value of effective communication within the realm of PIF. Communication skills such as active listening and open-ended questioning improve productivity and teamwork and promote trust among healthcare professionals, ultimately leading to improved patient outcomes. The emphasis placed on each of these qualities within each curriculum was estimated on a scale of one to four, with four indicating the greatest amount of importance and one indicating less importance. For physicians, communication is vital to establish trust and rapport with patients. Effective communication helps medical students and physicians internalize the values and behaviors expected of them, which helps them foster a sense of PIF when practicing [20]. In the realm of pharmacy, communication is crucial to pharmacists because of its necessity to prevent medication errors and user errors by patients. Pharmacists consider the task of dispensing medications properly as a crucial responsibility, which is why effective communication is highly valued in their professional identity [27].

With regard to responsibility, this core value of professional identity may be lacking a larger emphasis in pharmacy and nursing curricula due to the burden of patient outcomes being placed more on physicians and dentists as the leaders of the healthcare team. For pharmacists, the evolving nature of the profession leaves gaps in the embodiment of responsibility. Historically, pharmacists were not seen as the core of patient care, which can add to why they do not see responsibility as their core value [25]. For nurses, having high patient volumes, inadequate staffing, and insufficient support at times from the healthcare system can make it difficult for nurses to truly develop responsibility with patients. Additionally, nurses are usually given mentors from other staff members, which can make it also difficult to have a complete sense of responsibility [24]. These two reasons can add to why nurses do not have as much of a sense of responsibility in their core values. However, emphasizing shared responsibility in nursing and pharmacy education can help improve outcomes as these members of the team can take more ownership in the care of patients.

As for accountability, this core value is less emphasized in the pharmacist and nursing schooling system due to the inherent leadership role that physicians and dentists play in the healthcare team. Physicians and dentists tend to have more say in the decision making for patients in the hospital course or treatment options for patients. This can add to why physicians and dentists feel like they need to have a very high sense of accountability when it comes to their professional identity with their respective professions [28,29]. With this being said, both pharmacists and nurses also do have a sense of accountability but just not as high. However, shared or equal accountability among all team members can improve advocacy for their patients.

Communication is minimized as a core value in dental education due to the inherent separation of oral health and overall medical health. However, given the potential overlap between oral and other medical conditions, communication must receive stronger emphasis in dental education, which may be possible through interprofessional collaboration. The reduced significance that empathy receives in pharmacy schooling may be due to the inadequate training for patient education and key counseling that the physicians provide during their clinical encounters. To remedy this, pharmacy schools ought to further incorporate patient education and counseling activities in the curriculum for the development of a meaningful patient-pharmacist relationship.

Within an increasingly complex healthcare system, there is a heightened need for medical professionals to collaborate, value, and respect each other's roles that are shared by their experiences and expertise. Medical schools emphasize merging and accomplishing goals to improve patient outcomes through IPE [30]. According to the World Health Organization, IPE is “when two or more professions learn about, from, and with each other to enable effective collaboration and improve health outcomes” [5]. Learning with and from other health professional students in classes, simulations, and small groups encourages collaborative learning experiences within the healthcare profession [31]. These experiences prepare students for the workforce when individuals are required to collaborate with their colleagues to improve patient experiences [32].

Numerous reports indicate the critical role of IPE in PIF. Researchers have interviewed students of dentistry, medicine, nursing, pharmacy, and physiotherapy at the Atlantic Canadian University, five programs in the context of IPE and PIF [8]. The results indicated that the students who received IPE training were heavily focused on developing an IPE as opposed to a single professional identity. The students who had not received IPE yet had a limited understanding of their professional role and largely focused on individual identity development. The students with IPE training indicated a higher need for professional practice socialization. Various healthcare professional schools heavily emphasize this as a shared competency for PIF [33]. Health organizations have identified IPE in professional development to benefit the overall healthcare workforce in addition to interprofessional relationships and patient outcomes [34]. In another 2018 study, the IPE helped graduates develop increasing responsibility, practice communication skills, increase collaboration among different fields, and improve trust among coworkers [35]. Collaboration, responsibility, communication, and trust were all shared aspects of PIF across physicians, dentists, pharmacists, and nurses, as indicated in the table above. Based on these conclusions, it is evident that IPE can play a significant role in PIF, and all healthcare professional schools should provide opportunities and training in IPE and collaborative practice.

Our analysis has certain limitations. There is not enough published literature regarding PIF from nurses and pharmacists compared to physicians and dentists. This discrepancy makes it clear that pharmacists and nurses must be included more in the realm of PIF discussions in the future. It will allow a better understanding of the shared values and differences in PIF among various healthcare professions. Additionally, there is a lack of discussion on allied health professions, such as nurse practitioners and physician assistants regarding PIF and IPE. Future research is needed to explore further differences in how IPE between nurses and nurse practitioners and physician and physician assistants affect the PIF development of all health professionals.

## Conclusions

PIF is a crucial element in the development of healthcare learners to become competent and compassionate professionals in their respective fields. The process of PIF allows students to determine which core values are important for them to develop to become competent in their respective fields. The students are trained to develop a set of values through various patient interaction experiences with IPE, collaboration in their curriculum, and experiential learning. Based on the current literature, trust, collaboration, and empathy are the most highly regarded characteristics among a wide range of healthcare disciplines. Additionally, medical and dental students tend to place greater emphasis on responsibility and accountability as the final decision-making role in both professions.
